# Health Outcomes Associated with Loneliness and Social Isolation in Older Adults Living with HIV: A Systematic Review

**DOI:** 10.1007/s10461-024-04471-3

**Published:** 2024-09-04

**Authors:** Chava Pollak, Kelly Cotton, Jennifer Winter, Helena Blumen

**Affiliations:** 1 Department of Medicine (Geriatrics), Albert Einstein College of Medicine, 1225 Morris Park Ave, Bronx, NY 10461 USA; 2 Department of Neurology, Albert Einstein College of Medicine, 1225 Morris Park Ave, Bronx, NY 10461 USA; 3 College of Health Professions, Pace University, 861 Bedford Rd, Pleasantville, NY 10570 USA

**Keywords:** Aging, Older adults, Loneliness, Social isolation, HIV

## Abstract

Self-management of HIV is crucial to reduce disease-related negative health outcomes. Loneliness and social isolation are associated with poor disease self-management (e.g., medication non-adherence and care disengagement) in younger people with HIV and negative health outcomes in the general older adult population. Older adults with HIV (OWH) are at increased risk for loneliness and social isolation, but the associated health outcomes remain unclear. A comprehensive review of Pubmed, Embase, PsycINFO, and Web of Science databases was conducted. Criteria for inclusion were original quantitative research, published in the English language, included adults with a mean age ≥ 50 years, and included a measure for loneliness or social isolation, and a health outcome measure. A total of 41 studies were analyzed and 19,282 participants contributed to this review. The main findings were that loneliness and social isolation were associated with negative health behavior, disease self-management, physiological, and psychological outcomes. Pooled prevalence of loneliness was 33.9% across 8 studies. Loneliness and social isolation are highly prevalent in OWH and are associated with negative outcomes in OWH, similarly to older adults in the general population. Older adults with HIV, however, are challenged by unique psychosocial circumstances that place them at greater risk for loneliness and social isolation and associated negative health outcomes. These findings should be verified in larger, diverse, and longitudinal samples to better understand interrelationships of psychosocial factors and clinical outcomes in OWH.

**Supplementary Information:**

The online version contains supplementary material available at 10.1007/s10461-024-04471-3.

## Introduction

In the era of antiretroviral treatment (ART), the life expectancy of older adults with HIV (OWH) is largely comparable to individuals without HIV [1]. Over 50% of the 1.2 million people with HIV (PWH) are aged 50 and over [2]. HIV is a disease of accelerated aging and OWH commonly present with age-related comorbidities earlier than those without HIV due to immune system adaptations, chronic inflammation, and polypharmacy [3–7]. Loneliness and social isolation are highly prevalent in the general older adult population and are associated with poor health outcomes including increased risk of depression, cognitive and functional decline, dementia, hospitalization, and mortality [8–12]. Social isolation is defined as objective isolation from others including level of social engagement and social support, and structural measures such as living alone, number of social contacts, and relationship status [13]. Loneliness is defined as a subjective emotional state commonly described as a negative feeling of dissatisfaction with the quality or quantity of social relationships [13]. The two are overlapping yet distinct constructs because not everyone who is alone is lonely and not everyone who is lonely is alone [14]. Therefore, both phenomena must be considered together for a full picture of actual and perceived isolation.

OWH are particularly vulnerable to loneliness and social isolation due to a confluence of complex factors including HIV-related stigma—defined as prejudice, discrimination, or mistreatment used by individuals or societies to sanction people with HIV [15], restricted social networks, and reduced social engagement [15–17]. The combination of multimorbidity and accelerated aging observed in HIV, along with an increased risk for loneliness and social isolation, underscores the compounded risk for poor health outcomes in this uniquely vulnerable population. As the population of PWH continues to age, understanding clinical outcomes associated with loneliness and social isolation in this population is paramount.

In adults with HIV under aged 50, loneliness and social isolation are associated with substance use [18, 19], risky sexual behaviors [19], ART non-adherence [20], decreased engagement in care [21], cognitive decline [22], increased inflammation [23], and frailty [3]. Greater social support is associated with better physical function, better psychological well-being, lower self-reported HIV-related stigma [24, 25], and increased initiation of ART and engagement in care in both younger and middle-aged PWH [26]. However, research investigating loneliness and social isolation in OWH and the related health implications is scarce. Previous systematic reviews have focused on understanding the influence of psychosocial factors on aging in older women with HIV [27], assessing psychosocial interventions for OWH [28], and describing social networks in urban vs rural OWH [29]. To our knowledge, there are no prior systematic reviews synthesizing health outcomes associated with loneliness and social isolation in OWH, despite the increased prevalence of loneliness and social isolation in this growing population. The purpose of this review was to (1) quantify the burden of loneliness and social isolation in OWH, (2) synthesize the available literature on health outcomes associated with loneliness and social isolation in OWH, and (3) highlight areas for future research to better understand the impact of psychosocial well-being on health in this population.

There are multiple physiological and socio-psychological pathways through which social relationships might influence health outcomes, one of which is through health behaviors that influence morbidity and mortality [30]. Psychosocial risk factors such as loneliness and social isolation influence self-management of chronic disease and poor disease self-management leads to negative health outcomes [20]. According to the social control theory, individuals are influenced by those around them through modeling of positive health behaviors and reinforcement of health behavior norms [30]. This is especially true for intimate social ties such as marital and family relationships [30]. These findings are consequential for OWH who were living with HIV during the HIV/AIDS epidemic of the late 1970s and 1980s and HIV-related stigma may have affected family ties, especially for individuals who declined to disclose their HIV status [31]. Two key aspects of HIV self-management are medication adherence and care retention and individuals who are isolated or have low social support are less likely to follow up with care and adhere to medication regimens [16, 32]. The social control theory was used to conceptualize this review to understand how loneliness and social isolation might influence both health behaviors and health outcomes. Given that OWH are at high risk for loneliness and social isolation, understanding the influence of social relationships on all aspects of health in this population is important to magnify where and how to intervene.

## Methods

### Design

This systematic review was guided by the Preferred Reporting Items for Systematic Reviews and Meta Analyses (PRISMA) [33]. We evaluated studies that measured associations between loneliness and social isolation and clinical outcomes including health outcomes, healthcare utilization and retention, disease self-management, and health behaviors in OWH. The review was registered in PROSPERO (Registration #505234). For this review, we defined loneliness by any tool assessing perceptions of social isolation including one-item loneliness questions and other validated tools. Social isolation was defined as objective detachment or low contact with others including low social interaction/engagement, social network size, marital/relationship status, or living arrangements.

### Inclusion/Exclusion Criteria

Inclusion criteria were (1) quantitative research studies, (2) pertaining to community-dwelling adults aged 50 and over with HIV, (3) that include health outcome measures (e.g., physiological or psychological health conditions, healthcare utilization and retention, disease self-management, and health behaviors), and (4) are published in the English language. Given that HIV is a disease of accelerated aging, we included middle-aged as well as older adults rather than the commonly used age cut-off of 65 years [3, 4]. If samples were mixed (young, middle, and older adults), we included samples with a mean age of 50 and up. Exclusion criteria were the following: (1) qualitative studies, systematic reviews, dissertation studies, conference abstracts, study protocols, commentaries, measurement tool use or development studies, (2) did not include health outcome measures, or (3) did not include a loneliness or social isolation measure.

### Search Strategy

**Fig. 1:**
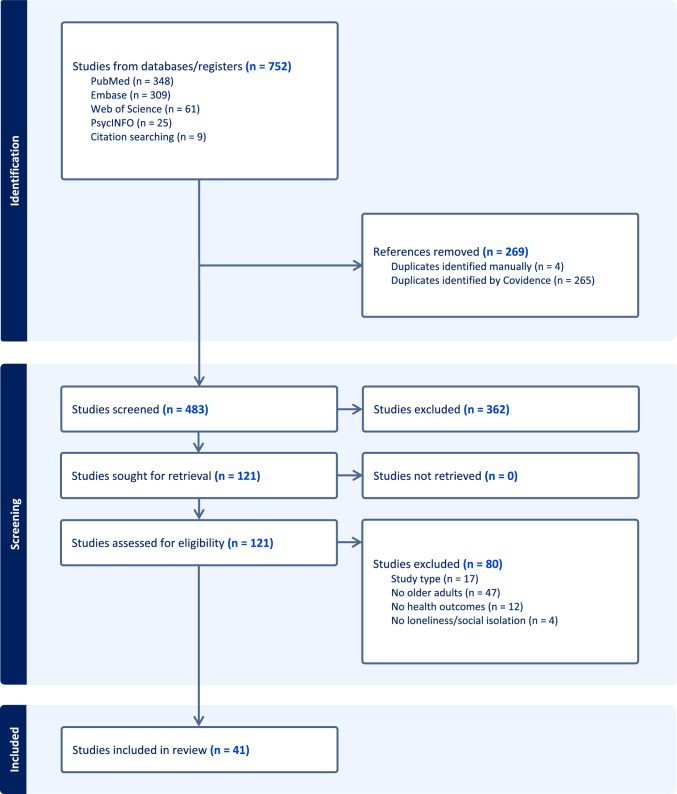
Flowchart of search strategy

A comprehensive search of electronic databases (Pubmed, Embase, PsycINFO, and Web of Science) was conducted in January 2024 by two authors (CP and KC) with no limit on dates searched, targeting keywords for “loneliness or social isolation,” “HIV,” and “Middle aged or older adult.” A full list of search terms used is enumerated in the supplementary material (eTable 1). Reference lists of included articles were hand-searched for additional relevant publications based on inclusion/exclusion criteria. A flowchart of the study search strategy is displayed in Fig. 1. The preliminary search identified 751 articles. After 269 duplicates were removed, 482 articles remained for initial screening, 362 of which were excluded. Out of 120 articles extracted for full text review, 80 were excluded: 47 did not include older adults, 17 included non-quantitative studies, 12 did not include a clinical outcome, and 4 did not include a loneliness or social isolation measure. Nine additional studies were identified through a hand search of references. A total of 41 studies evaluating associations between loneliness or social isolation and health outcomes in older adults with HIV were included in the final analysis.

### Data Collection/Analysis

Search results from the 4 databases were imported into Covidence software for screening and extraction. Titles and abstracts and full texts were screened by two reviewers (CP and KC). Disagreements were resolved via discussion. Data was extracted by two reviewers (CP and KC) and discrepancies were resolved by consensus. For each article that met all inclusion criteria, the following data was extracted: Year and country of publication, study design, purpose of the study, sample characteristics, loneliness/social isolation measures, health outcome measures, and pertinent main findings, including proportion of individuals who reported feeling lonely, as available (Table 1). All articles were assessed for quality using Joanna Briggs Institute critical appraisal tools (Joanna Briggs Institute, 2020. *JBI Critical Appraisal Checklist for Cohort Studies*) by two authors independently (CP and JW; see Supplementary Material eTable 2 and Section A for details).

**Table 1:** Summary of included literature: health outcomes associated with loneliness and social isolation

Citation, country of publication	Study design	Purpose of the study	Sample characteristics	Loneliness/social isolation measure	Health outcome measures	Main findings
Brouillette et al. (2022), Canada	Interrupted time series	To estimate the time course of psychological distress over the first wave of the COVID-19 pandemic and the associations between risk and resilience factors and distress	n = 77 90% Male Race not reported Mean age 57.3 Mean duration of HIV infection: 20.1 years	Living alone, no one to confide in, low social interaction OARS Loneliness item—feels lonely quite often	Hospital Anxiety and Depression Scale Connor-Davidson Resilience Scale	Feeling lonely or not having someone to confide in was associated with psychological distress
Derry et al. (2022), United States	Cross-sectional	To investigate cross-sectional relationships between psychosocial factors, inflammatory markers, and age-related health outcomes (self-rated function, falls, frailty)	n = 143 68% Male 50% African American Mean age 61 Mean duration of HIV infection: 23.2 years	13 item UCLA Loneliness Scale	Medical Outcomes Study-HIV Health Survey (quality of life) Fall in the last 6 months Fried frailty phenotype	Loneliness was not significantly associated with inflammatory factors. Sex or age did not moderate this relationship
Drewes et al. (2021), Germany	Descriptive, cross-sectional	To describe fall prevalence in community dwelling older adults with HIV and evaluate factors associated with fall risk	n = 897 87.1% Male Race not reported Mean age 57 Mean duration of HIV infection: 17 years	OSLO 3 Social Support Scale (OSSS) 3-item UCLA Loneliness Scale	Number of falls in previous 12 months	More social support was associated with reduced odds of falling. More feelings of loneliness and living alone were related to an increased odds for falling
Earnshaw et al. (2015), United States	Cross-sectional	Examine whether social support, adaptive coping, and/or HIV identity centrality moderate the impact of enacted and/or anticipated stigma on HIV symptoms	n = 93 55% Male Race: 52% Black 18% White 17% Other 36% Latino Age: mean 50.28 Mean duration of HIV infection: 18.1 years	Modified Medical Outcomes Study Social Support Survey Perceived community support (access to services)	Revised Sign and Symptom Check-List for HIV	Instrumental (but not emotional) social support and perceived community support acted as resilience resources for associations between stigma and HIV symptoms. At low levels of support, stigma was associated with HIV symptoms via stress; at higher levels of support stigma was not associated with HIV symptoms via stress
Eaton et al. (2020), Tanzania	Cross-sectional	To explore risk factors for symptomatic HIV-associated neurocognitive disorder (HAND) in HIV positive individuals aged 50 and over in Tanzania	N = 253 72.3% Female Race not reported Mean age 57.9	Living alone	Comprehensive neurocognitive assessment battery	Living alone was associated with a greater risk of symptomatic HAND
Emlet et al. (2013), United States	Cross-sectional	To identify risk and protective factors associated with mental and physical health-related quality of life	n = 226 100% male 77% non-Hispanic White Mean age 62.97	4-item Social Support Instrument	SF-8 Health Survey (physical and mental health related quality of life)	Social support significantly predicted mental health related quality of life but not physical health related quality of life
Emlet et al. (2017), United States	Cross-sectional	To examine HIV-related factors, adverse experiences, and psychosocial characteristics that are associated with resilience and mastery in HIV-positive gay and bisexual older men	n = 335 100% male 68% White, 14.6% Hispanic, 15% African American Mean age 58 Mean duration of HIV infection: 20 years	4-item Medical Outcomes Study Social Support Scale (MOS-SSS) Engagement in LGBT community—4 item scale (I help other people in the community, I am active or socialize in the community)	World Health Organization Quality of Life-BREF (WHOQOL-BREF) Resilience—3 item scale (I tend to bounce back quickly)	Social support and community engagement were positively associated with both resilience and mastery, which were separately associated with HRQOL
Emlet et al. (2020), United States	Cross-sectional	To examine whether disparities exist in poor health and depressive symptomatology among older gay/bisexual men aged 50 and older with and without HIV and identify risk and protective factors for those disparities	n = 1344 (371 HIV+) 100% male 59.6% Non-Hispanic White (HIV+), 87.3% (HIV−) Mean age 58.2 (HIV+), 63.4 (HIV−)	4-item Medical Outcomes Study Social Support Scale 4-item Community Engagement Scale Relationship status	Self-rated poor general health Center for Epidemiologic Studies Depression Scale (CESD-10)	Low social support and low LGBT community engagement was associated with self-rated poor general health and depressive symptomatology. Social support and LGBT engagement mediated the relationship between HIV+ status and poor general health and depressive symptomatology
Enel et al. (2019), France	Cross-sectional	To investigate the relationship between social deprivation, HIV status and health-related risk factors, physical and mental functioning in adults aged 50 and older	n = 494 72.9% Male Race not reported Mean age 58.5 Mean duration of HIV infection: > 25 years in 21.9% of participants	Evaluation of Deprivation and Inequalities in Health Examination Center Score (EPICES)	Katz Index for Activities of Daily Living 4-item Geriatric Depression Scale (GDS) History of falls or fracture (last 6 months) Visual Analogue Scale (VAS) for chronic pain Fried frailty phenotype	Social deprivation was associated with substance use (alcohol, tobacco, drugs), depression, falls or fractures, ADL disability, frailty, and presence of chronic pain
Golub et al. (2010), United States	Cross-sectional	To investigate the prevalence and correlates of sexual behavior among HIV positive adults over age 50	N = 914 70% Male 50% African American; 32.7% Latino; 12.8% Caucasian Median age 54	3-item UCLA Loneliness Scale	Sexual risk behavior measured by unprotected sex	Loneliness was associated with decreased odds of sexual activity and increased odds of unprotected sex in past 3 months
Greene et al. (2018), United States	Cross-sectional	To identify the prevalence of loneliness among older adults living with HIV, examine characteristics of lonely OLWH, and examine the association of loneliness with functional impairment and health related quality of life (HRQoL)	n = 356 87% Male 6% Latino, 43% non-White race Mean age 56 Mean duration of HIV infection: 50% > 20 years	8 item UCLA Loneliness Scale Lubben Social Network Scale	Self-rated health (poor, fair, good, very good, excellent) Lawton Instrumental Activities of Daily Living Scale Veterans Aging Cohort Study (VACS) index Patient Health Questionnaire (PHQ-9) Scale	Lonely participants were more likely to be smoke, drink alcohol, and use drugs, had increased depressive symptoms, lower self-rated health, IADL disability, and higher comorbidities
Greysen et al. (2013), United States	Observational, longitudinal cohort study	Investigate the prevalence and effects of social isolation on inpatient admission and outcomes of care in HIV+ older adults compared to HIV− older adults	n = 1836 (847 HIV+) 99% Male 68% non-white/Hispanic, 54% (HIV+) Mean age 61	Social Isolation Score (including engagement, marital status, living alone)	Hospital admission All-cause mortality	Social isolation was independently associated with increased risk of incident hospitalization and all-cause mortality
Grov et al. (2010), United States	Descriptive, cross-sectional	To assess the role of perceived health, stigma, and loneliness in predicting depression in community dwelling older adults	n = 914 70% Male 49.8% African American; 32.7% Latino; 12.7% White Median age 54 Mean duration of HIV infection: 12.6 years	20-item UCLA Loneliness Scale	Center for Epidemiological Studies Depression Scale (CES-D) Medical Outcomes Scale—HIV	Loneliness significantly increased the odds of depressive symptoms. Loneliness was not significantly associated to CD4 count or AIDS diagnosis
Guaraldi et al. (2022), Italy	Cross-sectional	To characterize frailty and resilience in PLWH and effects on health related quality of life (HRQoL)	N = 575 77.6% Male Race not reported Mean age 54.5 Median duration of HIV infection: 24.3 years	SUNFRAIL screening questionnaire (Do you feel lonely most of the time; Can you count on someone close to you in case of need)	37-item Frailty Index Connor Davidson Resilience Scale (CD-RISC-25) EQ-5D5L questionnaire Short Form 36 (SF-36) Health Survey Questionnaire	Loneliness predicted impaired resilience. Social support was protective against impaired resilience
Han et al. (2017), United States	Cross-sectional	To test the hypotheses that older Black adults with HIV would show greater loneliness than older White adults with HIV and that greater loneliness among older Black adults with HIV would be associated with poorer cognitive function	n = 370 (177 HIV+) 76% Male 70% Black; 30% White Mean age 58.7	de Jong-Gierveld Loneliness Scale	Complex Ideational Material and Mini-mental State Exam for diagnosis of cognitive impairment and description Battery of 19 cognitive measures	Older Black adults with HIV reported less loneliness than older White adults. In older Black (but not White) adults with HIV, greater loneliness was associated with lower global cognition
Han et al. (2021), China	Cross-sectional	Examine the factors that mediate the association between perceived discrimination and symptoms of cognitive dysfunction	n = 321 64.8% Male Race not reported Mean age 55.7 Mean duration of HIV infection: 4.3 years	3-item social functioning subscale of the AIDS Health Assessment Questionnaire	cognitive dimension of the WHO Quality of Life HIV Instrument (WHOQOL HIV)	Social isolation was significantly associated with more severe SOCD. Perceived discrimination influence symptoms of cognitive dysfunction via social isolation
Harris et al. (2020), Canada	Cross-sectional	To estimate the extent to which loneliness has associations with characteristics of the individual, environment, brain and general health, and quality of life among middle-aged and older adults living with HIV in Canada	n = 834 85% Male 73% White Mean age 53 Mean duration of HIV infection: 16.2–17.6 years	Older Americans Resources and Services (OARS) Social Resource Scale item 5 (do you find yourself feeling lonely often/sometimes/almost never)	RAND-36 WHOQOL-HIV BREF Perceived Deficits Questionnaire (PDQ—cognition) EuroQol EQ-5D-3L Patient Generated Index (PGI—QOL) Trier Inventory for Chronic Stress (TICS) Hospital Anxiety and Depression Scale (HADS) WHO5 Well-Being Index (mood)	HIV-related variables were not significantly associated with loneliness. Loneliness was associated with increased odds of cognitive impairment, low mood, stress, anxiety, depression, poor physical health, opioid use, and poor self-rated health and quality of life
Herbert et al. (2022), United States	Ecological Momentary Assessment Study	To examine the relationship between social contact frequency and pain, and the role of positive and negative affect in this relationship in older adults with HIV	n = 66 80.3% Male 63.6% White; 22.7% Black; 10.6% Hispanic Mean age 59.3 Mean duration of HIV infection: mean 22.8 years	Social contact frequency	Pain (what is your pain level right now)	More frequent social contact was associated with lower pain and greater pain was associated with lower subsequent social contact. Social contact frequency weakened associations between negative affect and pain
Hussain et al. (2023), United States	Cross-sectional	To investigate the relationship between loneliness and inflammation, and the effects of loneliness and inflammation on depression in PLWH	n = 82 86.6% Male 58.5% White, 19.5% Hispanic, 13.4% Black Mean age 53.2 Mean duration of HIV infection: 16.3 years	20-item UCLA Loneliness Scale 4-item Duke Social Support Index	Center for Epidemiologic Studies Depression Scale (CESD) Inflammatory biomarkers (CRP, IL-6, CCL2/MCP-1, sCD14, D-dimer)	Loneliness was significantly associated with higher levels of D-dimer, but not other inflammation biomarkers. Loneliness was associated with increased depressive symptoms
Kamalyan et al. (2021), United States	Ecological Momentary Assessment Study	To assess time spent at home among older adults with and without HIV using GPS data and to examine real-time relationships between life-space, mood (happiness, sadness, anxiety), fatigue, and pain among older adults and if these relationships were moderated by HIV serostatus	n = 88 61% HIV+ 82% Male (HIV+); 53% (HIV−) 65% White; 22% Black (HIV+) 18% (HIV−); 8% Hispanic (HIV+) 12% (HIV−) Mean age 60 Median duration of HIV infection: 25.3 years	Social contact frequency via EMA and time spent at home via GPS	Mood, pain, and fatigue via EMA	OLWH were more likely to have lower happiness, live alone, and spend more time at home compared to HIV− older adults. Associations between time spent at home and happiness were explained by social interactions with others in HIV+ and HIV− groups
Mannes et al. (2016), United States	Cross-sectional	To test associations between loneliness and substance use and whether the association was moderated by gender in HIV+ older adults	n = 96 63% Female 96% African American Mean age 56 Mean duration of HIV infection: 14.8 years	20-item UCLA Loneliness Scale	Substance use (self report and urinalysis)	Loneliness predicted substance use (marijuana) and heavy drinking in women, but not men
Mannes et al. (2017), United States	Cross-sectional	To examine the influence of age on associations between affective states, social support, and alcohol use by age categories	n = 96 62.9% Female 100% Black Mean age 55.8 Mean duration of HIV infection: 14.8 years	20-item UCLA Loneliness Scale ENRICHD Social Support Instrument (ESSI)	Alcohol consumption (self report)	In adults aged 50–59 and aged 60 and older loneliness was not significantly associated with alcohol consumption. In adults 60 and older, social support was associated with less alcohol use
Mayo et al. (2022), Canada	Longitudinal	Estimate the extent to which people aging with HIV meet criteria for successful aging as operationalized through HRQL and maintain this status over time and identify factors that place people at promise for continued successful aging, including environmental and resilience factors	n = 513 89% Male 77.8% White (male), 45.6% (female); 5.7% Black (male), 22.8% (female); Mixed 11.4% (male), 19.3% (female) Mean age 58 (male) 57 (female) Mean duration of HIV infection: 18.8 years (male), 16.5 (female)	RAND-36 (Do you find yourself feeling lonely? Do you have someone you trust and can confide in?; Time with someone who does not live with you?)	Successful aging (RAND-36—8 subscales)	The most influential factors associated with successful aging were loneliness and social network. 28.4% of people who report feeling "almost never" lonely were classified as successful aging, compared to 4.6% of those who reported feeling sometimes or often lonely
Mazonson et al. (2020), United States	Cross-sectional	To describe loneliness and its correlates in older adults living with HIV	n = 998 89% Male 69% White Mean age 59.4 Mean duration of HIV infection: 21 years	3-item UCLA Loneliness Scale	13-item PozQoL Scale PHQ-4 AUDIT-C (substance use)	Individuals who were lonely were more likely to report anxiety, depression, more comorbidities, current tobacco use, and lower quality of life, but not current recreational drug use. Logistic regression showed association between risk of loneliness and depression, recreational drug use, tobacco use, and quality of life
Meireles, et al. (2023), United States	Longitudinal Cohort Study	To assess the bi-directional association between frailty and loneliness over 2 years among sexual minority men living with and without HIV	n = 1118 (557 HIV+) 100% Male 57% White, 29%Black, 8% Hispanic (HIV+), 81% White, 12% Black, 5% Hispanic (HIV−) Median age 57 (HIV+), 62 (HIV−)	3-item UCLA Loneliness Scale	Fried Frailty Phenotype	Prevalence of loneliness was higher among PLWH compared to HIV− older adults. Loneliness at baseline predicted frailty at 2-year follow up. Frailty did not predict future loneliness
Moore et al. (2018), United States	Cross-sectional	To characterize successful cognitive aging among older HIV+ and HIV− older adults and to determine associations with positive psychological factors and HRQoL	n = 145 (99 HIV+) 79% Male 76% White Mean age 58.7 Mean duration of HIV infection: 17.6–18.2 years	Duke Social Support Index (DSSI) 2-item Emotional Support Scale	Successful Cognitive Aging defined by the absence of cognitive impairment, current MDD diagnosis, and IADL dependence	There was no difference in social support and other psychological factors and successful cognitive aging in HIV+ or HIV− older adults
Mwangala et al. (2022), Kenya	Cross-sectional	To determine the prevalence of depressive and anxiety symptoms among OLWH compared to HIV-negative peers, investigate HIV status as an independent predictor of depressive and anxiety symptoms, and investigate the determinants of common mental disorders in older adults in Kenya	N = 440 (257 HIV+) 58.6% Female Race not reported Mean age 60.1 Mean duration of HIV infection: 11.4 years	8-item UCLA Loneliness Scale	7-item Generalized Anxiety Disorder Scale (GAD-7) 9-item Patient Health Questionnaire (PHQ-9)	Loneliness was significantly associated with higher odds of anxiety but not depressive symptoms in OLWH
Nguyen et al. (2018), United States	Cross-sectional	To examine the risk and protective factors for Health-related quality of life (HRQOL) in older adults living with HIV	n = 176 75% Male 69.3% African American, 5.7% Hispanic/Latino, 25% White Mean age 58.7 Mean duration of HIV infection: 16.9 years	de Jong-Gierveld Loneliness Scale	Healthy Days Core of the CDC HRQOL Self-reported health Healthy Days Index (2-item)	Negative relationship between HDI and emotional loneliness. Loneliness not significantly associated with self-reported health status or healthy days index in linear regression model
Ogletree et al. (2019), United States	Cross-sectional	To assess the influence of multifaceted health burden indicators on depressive symptoms and evaluating the mediating effects of social support using the stress process model as a theoretical framework	n = 640 100% male 49.5% Black, 33.8% Hispanic, 16.7% White Mean age 55.7 Mean duration of HIV infection: 13.1 years	Instrumental social support (During the past year how much more help did you need with shopping, house cleaning, cooking, getting a ride) Emotional social support (During the past year, how much more emotional support (e.g., having someone to talk to or help make a big decision) did you need?)	Self-rated health (How would you rate your physical health at this time?) Self-reported HIV-related chronic conditions Age-related chronic conditions 10-item Center for Epidemiologic Studies Depression Scale (CES-D)	Instrumental support was associated with better self-rated health, emotional support was associated with HIV related conditions and self-rated health and both were associated with depressive symptoms. Perceived emotional, but not instrumental, support significantly mediated associations between health burden and depressive symptoms
Paolillo et al. (2018), United States	Ecological Momentary Assessment	To examine real-time relationships between social activity and mood, fatigue, and pain in a sample of older PLWH	n = 20 85% Male 70% White Mean age 58.8 Mean duration of HIV: 20.4 years	Social activity collected via EMA	Mood, fatigue, and pain collected via EMA	Being alone was associated with lower happiness and later-day fatigue and pain predicted greater likelihood of being alone later in the day. People were happier when not alone
Parish et al. (2020), United States	Cross-sectional	Examine how have unmet dental needs, as well as other demographic, behavioral, and clinical variables, affects the OHRQOL of women living with HIV	n = 1526 100% female 73.1% African American, 13.7% Hispanic, 10.4% White, 2.9% Other 52.6% aged 50 and over	3-item Loneliness Scale Sarason Social Support Questionnaire	Oral Health Impact Profile (OHIP-14)	Loneliness was significantly associated with worse OHRQOL
Petroll et al. (2023), United States	Descriptive, cross-sectional	To describe potential factors that may impact engagement in HIV care among older, rural-dwelling people living with HIV	n = 446 67% Male 67% White, 23% Black, 5% Multiracial, 5% Other race Mean age 55.6 Mean duration of HIV infection: 20 years	8-item MOS Social Support Scale Living alone Relationship status HIV support group attendance	Engagement in care (medications, appointments, viral load)	Individuals who lives alone had better engagement compared to those who lived with others. Relationship status, social support and engagement were not associated with engagement
Rendina et al. (2019), United States	Cross-sectional	To examine the effects of several psychosocial factors—depression, loneliness, alcohol and drug use, and HIV stigma—on ART adherence, viral suppression, and CD4 count in OLWH	n = 120 67.5% Male 75.8% Black, 9.2% Latino, 7.5% White, 7.5% Multiracial Mean age 54.6 Mean duration of HIV infection: 17.1 years	8-item UCLA Loneliness Scale	30 day TLFB interview for ART adherence Viral load suppression CD4 + cell count	Only HIV-related stigma was significantly associated with ART adherence and viral suppression; loneliness was not significantly associated with adherence or viral suppression and there was no statistically significant additive effect of psychosocial stressors
Rubtsova et al. (2021), United States	Cross-sectional	To test a psychosocial model of self-rated successful aging in OLWH	n = 356 100% Female 73.3% Black, 17.4% Hispanic, 6.2% White Mean age 56.5	3-item UCLA Loneliness Scale	Self-report "Using your own definition, where would you rate yourself in terms of successful aging from 1 to 10?"	Loneliness, along with anxiety, depression, and internalized HIV-related stigma in a composite measure of psychological distress was significantly associated with decreased self-rated health via decreased protective attributes and coping with stress
Siconolfi et al. (2013), United States	Cross-sectional	To characterize recent drug use of HIV+ adults aged 50 and older, examine differences in patters by heterosexual women, heterosexual men, and gay and bisexual men, and examine the relations between mental health status and substance use	n = 811 74% Male 50.9% Black, 32.3% Latino, 13% White Mean age 55.7 Mean duration of HIV infection: 12.7 years	20-item UCLA Loneliness Scale	Self-reported substance use in the prior 3 months	Loneliness was not significantly associated with substance use, potentially due to high prevalence of loneliness in the sample
Sun-Suslow et al. (2020), United States	Longitudinal Cohort Study	To investigate the influence of social support on the relationship between successful aging and D-dimer	n = 230 (134 HIV+) 75% Male (HIV+), 68% (HIV−) 53% White, 16% Black, 20% Hispanic, 2% Asian (HIV+), 68% White, 13% Black, 17% Hispanic, 0% Asian (HIV−) Mean age 51 Mean duration of HIV infection: 14.4 years	Duke Social Support Index (DSSI)	Self-rated successful aging on a scale from 1 to 10—"Using your own definition, where would you rate yourself in terms of successful aging?" D-dimer (chronic inflammatory marker)	In OLWH, social support moderated the relationship between D-dimer and successful aging in HIV+ but not HIV− older adults. The influence of social support on D-dimer and successful aging was most influential at the positive and negative extremes of social support in the older age cohort (ages 56–65 at baseline)
Vincent et al. (2017), United States	Cross-sectional	Investigated how HIV-related shame is associated with health-related quality of life in older people living with HIV	n = 299 67% Male 49.2% Black, 29.1% White, 8.7% Black Hispanic/Latino, 7% White, 2.3% Native American Mean age 55.2 Mean duration of HIV infection: 12.4 years	10-item UCLA Loneliness Scale	47-item Functional Assessment of Human Immunodeficiency Virus Infection (FAHI)	Loneliness mediated the relationship between HIV-related shame and social aspects of quality of life. Depression, but not loneliness, mediated the relationship between HIV-related shame and physical well-being
Wang et al. (2023), China	Cross-sectional	To describe prevalence and demographic correlates of loneliness in OLWH in China	n = 680 73.5% Male Race not reported Mean age 60.3 28% aged 50–54, 27% aged 55–59, 17.4% aged 60–64, 27.8% aged 65+	3-item UCLA Loneliness Scale	Self-perceptions of aging (Brief Aging Perceptions Questionnaire); Life satisfaction; functional ability (difficulty going upstairs or walking); comorbid conditions/multiple chronic conditions; alcohol or tobacco use	Loneliness was associated with higher self-perception of aging and less life satisfaction
Yoo-Jeong et al. (2020), United States	Cross-sectional	To identify factors related to loneliness in a sample of OLWH	n = 146 60.3% Male 85.6% Black, 8.2% White Mean age 56.5 Mean duration of HIV infection: 18.1 years	8-item Patient-Reported Outcomes Measurement Information System (PROMIS)—Social Isolation (SI)	Center for Epidemiologic Studies Depression Scale Revised (CESD-R) Charlson Comorbidity Index Lawton Instrumental Activities of Daily Living Scale	Comorbidity burden, functional status, and depressive symptoms were significantly associated with loneliness. Depression and HIV-related stigma explained 41% of the variance in loneliness, above and beyond the effects of unstable housing and disease burden
Yoo-Jeong et al. (2021), United States	Longitudinal	To test whether social isolation is related to retention in care directly or indirectly through emotional dysregulation in OLWH	n = 144 60.3% Male 85.6% African American, 8.2% White Mean age 56.6 Mean duration of HIV infection: 18.1 years	8-item Patient-Reported Outcomes Measurement Information System (PROMIS)—Social Isolation (SI) Social Network Index	Visit adherence over 12 month period	Loneliness and social network were not significantly associated with visit adherence. The indirect effect of emotion dysregulation on adherence through loneliness and social isolation was not significant
Yoo-Jeong et al. (2022), United States	Cross-sectional	To test the mediating effects of loneliness between stigma and depressive symptoms	n = 146 90% Male 85.6% Black Mean age 56.5 Mean duration of HIV infection: 18.1 years	8-item Patient-Reported Outcomes Measurement Information System (PROMIS)—Social Isolation	Center for Epidemiologic Studies Depression Scale Revised (CESD-R)	Loneliness mediated the association between HIV-related stigma and depressive symptoms

For the studies that included data relevant to loneliness prevalence, we conducted a random-effects meta-analysis to describe prevalence of loneliness across studies in older adults with HIV. Because prevalence is a proportion, we log-transformed the values. We assessed between-study heterogeneity with the Cochrane Q and I^2^ statistics. We used the meta package [34] in R statistical computing software v. 4.3.1 to calculate the pooled prevalence estimate and produce forest plots.

## Results

### Description of Studies

#### Study Design

Most studies were cross-sectional (n = 32) [35–66]; 5 were longitudinal [67–71], and 4 used other study designs (e.g., Ecological Momentary Assessment studies or interrupted time series) [72–75]. Included studies were published between 2010 and 2023; earlier studies from the late 1980s to early 2000s understandably included mostly younger adults (given that the HIV epidemic began in the late 1970s and individuals are now aging with HIV in the era of antiretroviral therapy).

#### Sample Characteristics

Most studies originated from the United States (n = 31). Other studies originated from Canada (n = 3), China (n = 2), Tanzania (n = 1), Kenya (n = 1), France (n = 1), Italy (n = 1), and Germany (n = 1). The total number of participants included in this review was 19,282 (although some studies used overlapping cohorts). Sample sizes ranged from 20 [75] to 1526 [53] participants. Several studies included participants with and without HIV (n = 8) [38, 39, 48, 59, 69–71, 74]. Most studies were majority male (n = 34) and studies that reported race/ethnicity (n = 33) included predominantly African American/Black or Hispanic participants. Most samples were middle-aged with a mean age range of 50–63 years. Mean duration of HIV infection for PWH ranged from 4.3 to 25.3 years.

#### Theoretical Basis

Several studies included theoretical/conceptual frameworks to contextualize the factors that might influence health outcomes in OWH. One model was the Health Equity and Promotion Model (HEPM) that incorporates a life-course perspective to understand the interrelationships of positive and negative experiences that converge to influence health and well-being in disadvantaged populations [38]. The authors highlight the essentialness of focusing not only on risk factors for health outcomes in OWH but also resilience factors that support health and well-being [38]. One study used the positive-aging framework to conceptualize aging with HIV and risk and protective factors that influence health-related quality of life even in the presence of chronic disease [52]. Following the same theme, the resilience theory—which posits that individuals can respond and adapt to challenging circumstances—was used to understand how a complex interplay of risk and protective factors interact to influence health outcomes [35]. Pearlin’s Stress Process Model was used to explain how psychosocial resources (e.g., social support) mediate the relationship between physical health burden and psychological well-being in the context of aging with HIV due to the cumulative HIV-related and age-related disease burdens [37]. The Theory of Loneliness contextualized the potential relationship between loneliness, social network, and retention of care to test the hypothesis that loneliness was associated with emotional dysregulation that lead to negative health behaviors, including lack of retention in care [68]. The Cognitive Affective Behavioral Model was used to explain the psychological implications of concealing a stigma and the cognitive reserve model explained how factors such as stigma, depression, anxiety, and social isolation contribute to changes in cognition [46]. The underlying themes of these frameworks emphasize that individuals possess both risk and protective factors that interact to influence health outcomes and highlight areas of opportunity for intervention to promote health.

#### Loneliness and Social Isolation Measures

There was little homogeneity in measurement tools used for loneliness and social isolation. The most commonly used tool for loneliness was the UCLA Loneliness Scale (n = 17) although studies varied from the number of items included in the scale ranging from 3 to 20 items. Other measures included a one item loneliness question (n = 4) and the de Jong Gierveld Loneliness Scale (n = 2). Social isolation measures included diverse measures. Several studies used the Medical Outcomes Study Social Support Scale (MOS-SSS; 4 items or 8 items; n = 4). The Patient Reported Outcomes Measurement Information System—Social Isolation (PROMIS-SI—which includes items from the UCLA loneliness scale) was used in 3 studies. Other measurement tools included the Duke Social Support Index (n = 3), the AIDS Health Assessment Questionnaire (n = 1), the OSLO-3 Social Support Scale (n = 1), ENRICHD Social Support Instrument (ESSI; n = 1), Lubben Social Network Index (n = 1), Sarason Social Support Questionnaire (n = 1), Evaluation of Deprivation and Inequalities in Health Examination Center Score (EPICES; n = 1), 4-item Community Engagement Scale (n = 1), 2-item Emotional Support Instrument (n = 1), single-item emotional or instrumental support questions derived from other questionnaires (n = 1), and composite measures of social isolation consisting of engagement measures, relationship status, and living arrangements (n = 1).

#### Prevalence of Loneliness and Social Isolation

Most studies did not include prevalence of loneliness or social isolation in their samples, although many cited the increased prevalence of loneliness and social isolation demonstrated by other studies in the conceptualization of OWH as a population at increased risk. One longitudinal study found prevalence of loneliness was more than 50% [57]. Individuals in the 50–59 age group were at higher risk of loneliness compared to those aged 60 and over (53.8% vs. 46.2%) [57]. Another longitudinal study reported loneliness was more prevalent in individuals with HIV compared to those without HIV at baseline and follow up (39.7% vs 31.6% at time 1 and 36.9% vs 34.3% at time 2) [70]. Another study found 58% of participants reported at least some symptoms of loneliness: 24% reported mild symptoms, 22% reported moderate symptoms, and 12% reported severe loneliness [64]. Another study of 1526 women, reported a 49.9% prevalence of loneliness [53]. Finally, another study reported 39.7% of participants were lonely [63]. All the abovementioned studies were US-based samples and assessed loneliness using the UCLA Loneliness Scale. Due to the heterogeneity of social isolation measures, prevalence of social isolation was difficult to define, however, some studies included objective social isolation prevalence based on living arrangements and relationship status. A Canadian study reported 39% of participants lived alone [73]. A US-based study reported, of 998 OWH, 33% were in a relationship, 69% lived alone, and 81% reported not having close friends. [57] Another study also reported 69% of 914 OWH lived alone [61]. Another US-based sample of OWH reported 65% lived alone [35]. Several small studies of less than 150 OWH with varied demographic makeups reported between 9.6 and 11% were married [43, 54, 62, 63] while another study of 226 men reported 34% were married [35]. In contrast, one China-based sample reported 67% of 321 participants were married [46]. Another China-based cohort of 680 OWH reported 56.8% were in a stable relationship, 29.4% lived alone, and 59.3% reported close relationships with their children [41]. Of 257 OWH from Kenya, 44% were married, 12.5% were living alone and 58.8% reported sometimes or frequently lacking access to social support [48].

### Meta-analysis

**Fig. 2:**
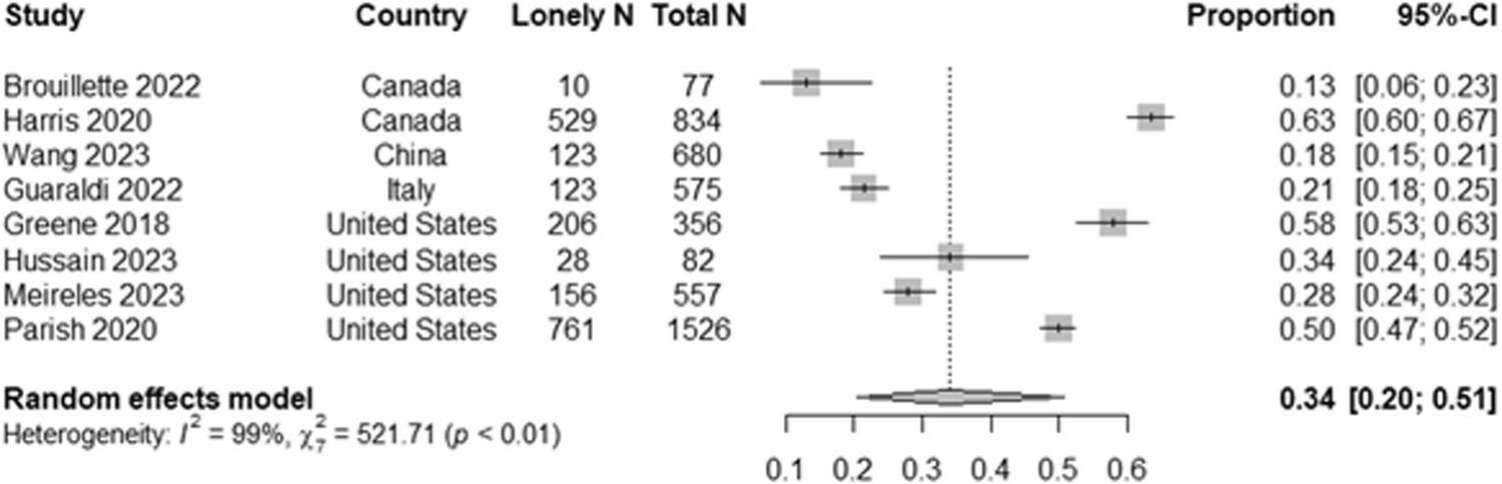
Forest plot of loneliness prevalence estimates from 8 included studies

Eight of the forty-one studies included relevant loneliness prevalence data (Fig. 2). Two of the studies used the Older Americans Resources and Services (OARS) Social Resource Scale loneliness question (“Do you find yourself feeling lonely often/sometimes/almost never?”), one study used the loneliness question from the European Sunfrail Network frailty tool (“Do you feel lonely most of the time?”), and five studies used the UCLA Loneliness Scale. Studies using the UCLA Loneliness Scale defined loneliness with different thresholds (8-item UCLA Loneliness Scale ≥ 17 [64], 20-item UCLA Loneliness Scale ≥ 44 [66], 3-item UCLA Loneliness Scale ≥ 6 [41, 70], and one study did not report the cut-off used for loneliness, however the authors use the 3-item UCLA Loneliness Scale) [53]. The pooled estimate loneliness prevalence among OWH was 33.9% (95% CI 20.4–50.8%). There was a substantial degree of heterogeneity (I^2^ = 98.7%, Q-test p < 0.001). This heterogeneity was likely due to differences in the loneliness measurements and cut-off values used or study sample characteristics.

#### Outcome Measures

Several different health outcomes were assessed. We included studies that directly investigated associations between loneliness and social isolation and health outcomes as well as studies that looked at correlates of loneliness and social isolation or risk factors for specific health outcomes and found associations for loneliness and social isolation. We organized the health outcomes into 4 categories: disease self-management, health behaviors, physiological health, and psychological health outcomes. We discuss the health outcomes in this order to align with the Social Control Theory of Behavior that guided this review. According to this theory, individual health behaviors and disease self-management (e.g., medication adherence, care retention) are influenced by the people around them, which in turn influence physiological and psychological health outcomes.

#### Health Behaviors

Social deprivation and loneliness were associated with risky sexual behaviors [51] and substance use in OWH including alcohol [42, 62, 64], tobacco [42, 57, 64], recreational drug use [42, 57, 62, 64], and opioid use [56], though one study found no association between loneliness and alcohol use [44]. Greater social support on the other hand, was associated with reduced alcohol use in adults aged 60 and older, but not those aged 50–59 years [43]. Studies investigating other health behaviors in OWH were not identified.

#### Disease Self-management

Only 3 studies examined associations between loneliness and social isolation and self-management outcomes. They showed loneliness, social support, social engagement, relationship status, and social network size were not associated with ART adherence [45, 55, 68], retention in care [55], or viral suppression [45, 55] and those who lived alone had better care engagement (medication adherence, retention in care, and suppressed viral load) compared to those who lived with others [55].

#### Physiological Health Outcomes

Social isolation was associated with increased risk for hospitalization and all-cause mortality [71]. Loneliness and social isolation were also associated with both cognitive [46, 49, 56, 59] and functional decline, including disability in activities of daily living (ADLs) [42] and instrumental activities of daily living (IADLs) [64], as well as frailty cross-sectionally [42] and at 2-year follow up [70]. Greater levels of social support were associated with reduced odds of falling and loneliness and living alone were associated with increased odds of falls [42, 47] and fractures [42]. Loneliness was also associated with the presence of chronic pain [56] and more frequent social contact was associated with lower pain [72]. Associations between loneliness and self-rated health were inconsistent; one study reported loneliness predicted poor self-rated health [56], while other studies found that loneliness was not associated with self-rated health [52] and social support was not significantly associated with physical health-related quality of life [35]. Finally, three studies reported mixed effects of loneliness and social isolation on inflammation. Social support buffered the relationship between chronic inflammation and successful aging, especially at older ages and extremes of social support [69] and loneliness was associated with increased inflammation [66], while another study found no association between loneliness and inflammatory markers [65].

#### Psychological Health Outcomes

Social support and community engagement were associated with resilience (using measures that assessed adaptation to challenges) [36, 50] and quality of life [35, 36] while loneliness was associated with impaired resilience [50] and reduced quality of life [53, 57], and reduced life satisfaction [41]. On the other hand, more social support mitigated the negative effects of HIV-related stigma and stress on HIV symptoms [40]. Loneliness and social isolation were associated with anxiety [48, 56, 57] and depressive symptoms [37, 38, 56, 57, 61, 64, 66]. However, reports on associations between loneliness and depressive symptoms in OWH were mixed. In several studies, loneliness was associated with increased depressive symptoms [56, 57, 61, 64] and the relationship between HIV-related stigma and depressive symptoms was mediated by loneliness [54]. In a sample of 257 mostly female OWH in Kenya, loneliness was not significantly associated with depressive symptoms [48]—discrepant findings that may be related to different characteristics of the samples (e.g., the Kenya sample was mostly married women, few of whom lived alone, while the Canadian and US samples were mostly single men, many of whom lived alone).

## Discussion

The purpose of this systematic review was to synthesize the available evidence on loneliness and social isolation on health outcomes in OWH and highlight future directions for research. Overall findings of the 41 included studies indicate loneliness and social isolation are highly prevalent in OWH and this population is uniquely vulnerable to loneliness and social isolation due to complex biopsychosocial factors associated with aging with HIV. The main findings included several health behavior, disease-self management, physiological, and psychological outcomes associated with loneliness and social isolation in OWH.

### Burden of Loneliness and Social Isolation in OWH

Studies that included individuals with and without HIV showed loneliness and social isolation were more prevalent in individuals with HIV [70]. Pooled prevalence of loneliness in OWH using data from 8 studies was 33.9%. Comparatively, prevalence of loneliness in the general older adult population across 29 countries is estimated at 28.5% [76]. Many studies did not report loneliness prevalence in their samples, which precluded inclusion of those data in the meta-analysis. Additionally, included studies reported OWH in the US more commonly lived alone and were uncommonly married compared to reports on OWH from studies in China, Canada, or Kenya. Most samples in this review were US-based samples and comparisons to other samples are therefore limited. However, in the general population in the US, an estimated 39% of older adults live alone [77] compared to close to 70% of OWH [35, 57, 61]. The demographic makeup of OWH and older adults in the general population may differ drastically and can be an important factor in these differences. The populations included in this review are largely represented by minoritized groups (e.g., racial/ethnic and sex/gender) who bear a heavy burden of social and socioeconomic disadvantage due to HIV-related stigma, related loneliness and social isolation, and morbidity. These data highlight the need for further research on the structural and functional psychosocial challenges experienced by OWH and associated health outcomes.

### Health Outcomes Associated with Loneliness and Social Isolation

The social control theory of behavior is generally supported by evidence from this review that loneliness and social isolation were linked with substance use and risky sexual behavior [42, 56, 57, 62, 64], however, these associations may differ across age groups [43]. Additionally, loneliness and social isolation were associated with a range of negative physiological and psychological outcomes. Since few studies investigated self-management and health behaviors, the relationship between social control as a mechanism for negative health outcomes associated with loneliness and social isolation remains unclear. A factor that was not explored in any of these studies was relationship quality which may be uniquely important in OWH. Not all relationships are positive and conflictual relationships may negatively influence health behaviors and outcomes [78, 79]. This is supported by a study that reported OWH who lived alone had better engagement in care compared to those who lived with others [55], suggesting either relationships may negatively impact care engagement, or an individual may be caring for an aging partner and not engaging in their own health or self-care. Additionally, HIV status disclosure, which may be influenced by relationship quality, is a crucial factor in terms of accessing social support and ability to take medications at home [16, 80, 81]. This highlights an important gap in the current literature base and points to an opportunity for future research to include not only the existence or number of relationships but also the quality of these relationships.

Many of the physiological and psychological outcomes associated with loneliness and social isolation in OWH were widely examined and well-established in the general older adult population including cognitive and functional decline, frailty, and depressive symptoms [9, 12, 82, 83]. Proposed biological mechanisms for associations between loneliness and social isolation and health outcomes include the inflammatory hypothesis, resilience theories, and impaired social cognition and emotional dysregulation, among others [84, 85]. These mechanisms are interconnected and uniquely applicable to OWH due to chronic inflammation associated with aging and HIV infection, impaired resilience related to multimorbidity and intersecting stigmas, and increased risk of loneliness and social isolation [3, 6, 7, 15, 23]. Additionally, accelerated aging related to HIV infection implies OWH may experience these health outcomes sooner compared to those without HIV. This is supported by representation of mostly middle-aged OWH samples in this review. The combination of HIV-related accelerated aging and increased morbidity and the risk of increased loneliness and social isolation in OWH compounds the risk for negative health outcomes in OWH and underscores the need to prioritize this population for intervention. Additionally, evidence for associations between loneliness and social isolation and discrete physiological outcomes including inflammatory markers [65, 69] and depressive symptoms [37, 48, 54, 61] in OWH were mixed and require further study to parse these relationships in this population.

A major limitation of the current body of research is the very small number of studies investigating self-management and health-behavior outcomes in OWH. Disease self-management and positive health behaviors (e.g., physical activity, sleep, medication management, care engagement [21, 26, 86–88]) are closely linked with loneliness and social isolation and both are crucial to understanding related physiological and psychological health outcomes. More studies are needed to understand self-management and health-behavior outcomes associated with loneliness and social isolation in OWH and barriers and facilitators to medication adherence, and care retention in this population. Additionally, studies on health behaviors of OWH are necessary to better understand behaviors associated with loneliness and social isolation that may be included in the planning and development of interventions.

Interestingly, most studies in this review did not find associations between loneliness and social isolation and self-management-related outcomes and one study reported those who lived alone had better engagement in care compared to those who did not live alone [55]. Additionally, evidence for emotional dysregulation as a mechanism for associations between social isolation and care retention in OWH was not established and requires further study [68]. The small number of studies on loneliness and social isolation and disease self-management, however, preempts conclusions on these findings. A study of younger and middle-aged PWH struggling with medication adherence and engagement in care found participants were isolated and lonely, lacked support from family and friends, and shouldered a heavy stigma burden [20], highlighting the challenges PWH may face and the role of social support and positive social relationships in disease self-management. A systematic review and meta-analysis showed depression and depressive symptoms were significantly associated with ART adherence [89]. Loneliness and social isolation are linked with depression in the general older adult population and OWH [90, 91], highlighting the importance of considering psychosocial factors in interventions targeting ART adherence. These findings underscore the need for further study in individuals aging with HIV as psychosocial factors may differentially influence medication adherence and engagement in care in older populations and mechanisms of action for these influences may differ across age groups and populations.

### The Role of HIV-Related Stigma

While this was not a review of outcomes associated with HIV-related stigma, it is impossible to discuss outcomes associated with loneliness and social isolation without considering the role of stigma. Stigma is defined as prejudice, discrimination, or mistreatment used by individuals or societies to sanction people with HIV and older adults with HIV may experience intersecting stigmas related to age, race, or gender identity at the same time [15]. Stigma in PWH influences both social behaviors and experiences, and negative health outcomes [15, 40, 92, 93]. Stigma was a running theme in many of the included studies, particularly regarding how stigma influenced loneliness and social isolation and thus resulted in poor health outcomes. Older OWH may experience intersecting HIV-related stigma and ageism that shape their experiences of living with HIV and contribute to inequities in health outcomes in this population [16, 93]. A better understanding of stigma experienced by OWH and the experience of loneliness and social isolation related to HIV-related stigma and other stigmas that may be experienced by OWH simultaneously (e.g., ageism, racism, discrimination based on gender identity) is necessary as the population of individuals with HIV is aging. Studies on loneliness and social isolation in OWH should include measures of HIV-related stigma to further examine these separate, but closely related constructs.

### Interventions for Loneliness and Social Isolation in OWH

The complexity of the care of OWH, combined with the intersection of multiple factors affecting their health, requires new approaches for intervention and underscores the significance of addressing the unmet needs of this population to include medical and psychosocial disparities. Inequities encountered by OWH include late diagnosis and entry to care due to lower perceived risk, higher rates of comorbidities impacting health outcomes, and sexual minority status [94]. An important challenge in terms of interventions for loneliness and social isolation for the OWH population is to address population-specific needs while also mainstreaming interventions for OWH in the general population to avoid perpetuating stigma and isolation [15]. Community connections can serve as important buffers to challenges of aging with HIV and several grassroots and advocacy initiatives were born to address these needs [94]. Additionally, technology can be leveraged to connect OWH in geographically isolated areas however, studies note challenges in terms of access and reliability [28, 94]. A systematic review of psychosocial interventions for OWH included a small number of studies and identified the need for further research on preventative interventions in this population [28]. Preventive interventions might include peer support groups, psychotherapy, technology-based interventions, education, and advocacy to address stigma and bias, and increased community engagement to influence policy [94].

### Strengths and Limitations

This review was conducted rigorously and provides a comprehensive synthesis of the current evidence on a wide variety of clinical outcomes of loneliness and social isolation in OWH. Results are limited by the small number of studies directly investigating health outcomes associated with loneliness and social isolation, small sample sizes, and small numbers of older adults (e.g., mean age in most samples were middle-aged). While most included studies included a mean duration of HIV infection of more than a decade, mean duration of HIV infection ranged from 4.3 to 25.3 years. Long-term survivors of HIV may represent a unique subset of older adults with HIV that bears further study. Most studies included US-based samples and representativeness from other countries is limited. Most studies that contributed loneliness prevalence data for the meta-analysis were from the US (n = 4) or Canada (n = 3), highlighting a need to report prevalence data for loneliness in future research. Additionally, heterogeneity in loneliness and social isolation measures as well as outcome measures precluded robust meta-analyses. Restriction of English-language only studies may have excluded international studies.

### Future Directions

We identified several areas for future studies of loneliness and social isolation in OWH. Importantly, many studies investigated relationships between loneliness and social isolation peripherally by identifying correlates and risk factors of loneliness and social isolation in OWH or by investigating loneliness as a mediator. While this establishes the importance of psychosocial factors and clinical outcomes, it precludes a nuanced understanding of their interrelationships. Further study is needed to examine the direct and indirect relationships between loneliness and social isolation and health behaviors, disease self-management, physiological, and psychological, outcomes in OWH to better understand how to intervene.

As individuals are aging with HIV, longitudinal studies are needed to understand the effects of loneliness and social isolation in this population over time. Longitudinal studies would also allow for comparisons between transient and persistent loneliness which may be associated with different health trajectories. Studies of older adults with and without HIV are needed to examine differences in health outcomes associated with loneliness and social isolation in older adults with and without HIV and determine if there is an interaction between HIV and loneliness and/or social isolation. Additionally, comparisons of health outcomes associated with loneliness and social isolation in newly infected older adults compared to those with HIV for many years would elucidate potentially discrete needs in these populations. Since medication adherence, care engagement, and viral suppression are bedrocks of HIV management, studies on psychosocial determinants of these outcomes specifically in OWH are needed to understand how best to intervene. The Social Control Theory may be used as a framework for future research to understand how social influences impact health behaviors and outcomes. Further studies including larger and more diverse samples that are inclusive of all individuals aging with HIV—including gender/sex and racial/ethnic minorities that bear a disproportionate HIV burden, adults in older age groups, OWH in rural environments who may have different challenges in terms of social isolation and access to care, and OWH across cultural contexts. Additionally, the demographics of OWH in different parts of the world and associated risk factors for loneliness and social isolation, including the experience of stigma, may vary across cultural populations [15] and deserve further study. The available studies in the US reported significantly higher prevalence of loneliness and social isolation compared to OWH in other countries. However, the limited number of studies from other countries hinders substantive comparisons. Consistent measures across studies would allow for meta-analyses comparing effects of loneliness and social isolation on clinical outcomes in older adults with and without HIV. Additionally, standardized cut-offs for loneliness would improve reporting and comparison of studies and clearly define loneliness as a phenomenon.

## Conclusions

Loneliness and social isolation are associated with negative health behavior, disease self-management, physiological, and psychological outcomes in OWH although mechanisms for these associations are unclear. OWH are at increased risk for loneliness and social isolation and should be prioritized for research and intervention. Findings should be verified in larger, culturally diverse populations in different ages and settings and samples should be followed longitudinally to evaluate effects over time.

## Supplementary Information

Below is the link to the electronic supplementary material.

Supplementary file1 (DOCX 29 KB)

Supplementary file2 (PNG 21 KB)
