# Supplementary material for: Health Outcomes Associated with Loneliness and Social Isolation in Older Adults Living with HIV: A Systematic Review
Source: AIDS Behav. 2024 Sep 4;29(1):166–86. doi: 10.1007/s10461-024-04471-3 (PMC11739194; doi:10.1007/s10461-024-04471-3)
Supplement: Supplementary file 1 — Supplementary file1 (DOCX 29 KB) [file 10461_2024_4471_MOESM1_ESM.docx]

**Supplementary Material**

eTable 1. Search Terms

eTable 2. Summary of critical appraisal of included literature

Section A. Critical appraisal of included literature

eFigure 1. Meta-analysis for loneliness prevalence by country

| **eTable 1. Search Terms by Database** | |
| --- | --- |
| **Database: PubMed** | |
| **Line #** | **Search Terms** |
| 1 | "Social Isolation"[Mesh:NoExp] OR "Loneliness"[Mesh] OR "Social Alienation"[Mesh] OR “Loneliness”[tiab] OR “Lonely”[tiab] OR “ Social Isolation”[tiab] OR “Social alienation”[tiab] OR “Social exclusion”[tiab] |
| 2 | "HIV"[Mesh] OR "HIV Infections"[Mesh] OR “Human immunodeficiency virus”[tiab] OR “HIV-positive”[tiab] OR “living with HIV”[tiab] |
| 3 | #1 AND #2 |
| 4 | #3 AND ("Middle Aged"[Mesh] OR "Aged"[Mesh] OR “middle age*”[tiab] OR “mid life”[tiab] OR “midlife”[tiab] OR “older adult*”[tiab] OR “older people”[tiab] OR “elder*”[tiab] OR “aging”[tiab]) |
| 5 | #4 NOT (“children”[tiab] OR “child”[tiab] OR “adolescent*”[tiab] OR “teenager*”[tiab] OR “young adult*”[tiab] OR “youth*”[tiab]) |
| **Database: Embase** | |
| **Line #** | **Search Terms** |
| 1 | 'social isolation'/de OR 'loneliness'/exp OR 'social alienation'/exp OR ‘Loneliness’:ti,ab OR ‘Lonely’:ti,ab OR ‘ Social Isolation’:ti,ab OR ‘Social alienation’:ti,ab OR ‘Social exclusion’:ti,ab |
| 2 | 'Human immunodeficiency virus'/mj OR 'Human immunodeficiency virus infection'/mj OR ‘Human immunodeficiency virus’:ti,ab  OR ‘HIV-positive’:ti,ab OR ‘living with HIV’:ti,ab |
| 3 | #1 AND #2 |
| 4 | #3 AND ('middle aged'/exp OR 'aged'/de OR 'frail elderly'/exp OR 'very elderly'/exp OR ‘middle age*’:ti,ab OR ‘mid life’:ti,ab OR ‘midlife’:ti,ab OR ‘older adult*’:ti,ab OR ‘older people’:ti,ab OR ‘elder*’:ti,ab OR ‘aging’:ti,ab) |
| 5 | #4 NOT (‘children’:ti,ab OR ‘child’:ti,ab OR ‘adolescent*’:ti,ab OR ‘teenager*’:ti,ab OR ‘young adult*’:ti,ab OR ‘youth*’:ti,ab) |
| **Database: PsycINFO** | |
| **Line #** | **Search Terms** |
| 1 | **Index terms**: {Social Isolation} OR {Loneliness} OR {Social Exclusion} OR {Social Deprivation} |
| 2 | **Title**: (“Loneliness” OR “Lonely” OR “ Social Isolation” OR “Social alienation” OR “Social exclusion”) OR **Abstract**: (“Loneliness” OR “Lonely” OR “ Social Isolation” OR “Social alienation” OR “Social exclusion”) |
| 3 | #1 OR #2 |
| 4 | **Index terms**: {Hiv} OR **Title**: (“HIV-positive” OR “living with HIV”) OR **Abstract**: (“HIV-positive” OR “living with HIV”) |
| 5 | **Title**: (“middle age*” OR “mid life” OR “midlife” OR “older adult*” OR “older people” OR “elder*” OR “aging”) OR **Abstract**: (“middle age*” OR “mid life” OR “midlife” OR “older adult*” OR “older people” OR “elder*” OR “aging”) |
| 6 | #3 AND #4 AND #5 |
| **Database: Web of Science** | |
| **Line #** | **Search Terms** |
| 1 | **Title**: (“Loneliness” OR “Lonely” OR “ Social Isolation” OR “Social alienation” OR “Social exclusion”) OR **Abstract**: (“Loneliness” OR “Lonely” OR “ Social Isolation” OR “Social alienation” OR “Social exclusion”) |
| 2 | **Title**: (“Human immunodeficiency virus” OR “HIV-positive” OR “living with HIV”) OR **Abstract**: (“Human immunodeficiency virus” OR “HIV-positive” OR “living with HIV”) |
| 3 | **Title**: (“middle age*” OR “mid life” OR “midlife” OR “older adult*” OR “older people” OR “elder*” OR “aging”) OR **Abstract**: (“middle age*” OR “mid life” OR “midlife” OR “older adult*” OR “older people” OR “elder*” OR “aging”) |
| 4 | #1 AND #2 AND #3 |
| 5 | **Title**: (“children” OR “child” OR “adolescent*” OR “teenager*” OR “young adult*” OR “youth*”) OR **Abstract**: (“children” OR “child” OR “adolescent*” OR “teenager*” OR “young adult*” OR “youth*”) |
| 6 | #4 NOT #5 |

| eTable 2. Summary of critical appraisal of included literature | | | | | | | | |
| --- | --- | --- | --- | --- | --- | --- | --- | --- |
| Article | Criteria for inclusion clearly defined | Study subjects and setting described in detail | Valid  loneliness or social isolation measure | Objective/  Standard criterion for HIV diagnosis | Confounding factors identified | Strategies to deal with confounding factors stated | Valid outcome measures | Appropriate statistical analysis |
| Brouillette et al. (2022) | X | X | X | X | X | X | X | X |
| Derry et al. (2022) | X | X | X | X | X | X | X | X |
| Drewes et al. (2021) | X | X | X | X | X | X | X | X |
| Earnshaw et al. (2015) | X | X | X | X | X | X | X | X |
| Eaton et al. (2020) | X | X | X | X | X | X | X | X |
| Emlet et al. (2013) | X | X | X |  | X | X | X | X |
| Emlet et al. (2017) | X | X | X | X | X | X | X | X |
| Emlet et al. (2020) | X | X | X | X | X | X | X | X |
| Enel et al. (2019) | X | X | X | X | X | X | X | X |
| Golub et al. (2010) | X | X | X | X | X | X | X | X |
| Greene et al. (2018) | X | X | X | X | X | X | X | X |
| Greysen et al. (2013) | X | X | X |  | X | X | X | X |
| Grov et al. (2010) | X | X | X | X | X | X | X | X |
| Guaraldi et al. (2022) | X | X | X |  | X | X | X | X |
| Han et al. (2017) | X | X | X | X | X | X | X | X |
| Han et al. (2021) | X | X | X | X | X | X | X | X |
| Harris et al. (2020) | X | X | X |  | X | X | X | X |
| Herbert et al. (2022) | X | X | X | X | X | X | X | X |
| Hussain et al. (2022) | X | X | X | X | X | X | X | X |
| Kamalyan et al. (2021) | X | X |  | X |  |  | X |  |
| Mannes et al. (2016) | X | X | X | X |  |  | X | X |
| Mannes et al. (2017) | X | X | X | X | X | X | X | X |
| Mayo et al. (2022) | X | X | X | X | X | X | X | X |
| Mazonson et al. (2020) | X | X | X | X | X | X | X | X |
| Meireles, et al. (2023) | X | X | X | X | X | X | X | X |
| Moore et al. (2018) | X | X |  |  | X | X | X | X |
| Mwangala et al. (2022) | X | X | X | X | X | X | X | X |
| Nguyen et al. (2018) |  | X | X |  |  |  | X | X |
| Ogletree et al. (2019) | X | X |  | X | X | X | X | X |
| Paolillo et al. (2018) | X | X |  | X |  |  |  | X |
| Parish et al. (2020) |  | X | X |  | X | X | X | X |
| Petroll et al. (2023) | X | X |  | X | X | X | X | X |
| Rendina et al. (2019) | X | X | X | X | X | X | X | X |
| Rubtsova et al. (2021) | X | X | X | X | X | X | X | X |
| Siconolfi et al. (2013) | X | X | X | X | X | X | X | X |
| Sun-Suslow et al. (2020) | X | X | X | X | X | X | X | X |
| Vincent et al. (2017) | X | X | X | X | X | X | X | X |
| Wang et al. (2023) | X | X | X | X | X | X | X | X |
| Yoo-Jeong et al. (2020) | X | X | X | X | X | X | X | X |
| Yoo-Jeong et al. (2021) | X | X | X | X | X | X | X | X |
| Yoo-Jeong et al. (2022) | X | X | X | X | X | X | X | X |

Section A. Critical appraisal of included literature

All studies were assessed for quality using the Joanna Briggs Institute Critical Appraisal tool by two evaluators (CP and JW). The Joanna Briggs tool utilizes checklist to assess quality and reliability of cross-sectional and longitudinal studies. The number of positive responses (referenced by the X marks in eTable 2) provides information regarding quality of the study. This process ensures a methodological approach to assess the quality of each study. Overall, the studies (N=41) were robust in most of the areas addressed in the appraisal tool. There were unclear aspects regarding the inclusion of a valid loneliness or social isolation tool in five studies. Six studies did not include standardized/objective criterion for the diagnosis or definition of HIV. Three studies were not found to have confounding factors or strategies to identify them. Only one study did not include an appropriate statistical analysis method. Overall, studies were of good quality and no studies were excluded based on quality to maximize use of available literature.
