# Supplementary figures and images for: Health Outcomes Associated with Loneliness and Social Isolation in Older Adults Living with HIV: A Systematic Review
Source: AIDS Behav. 2024 Sep 4;29(1):166–86. doi: 10.1007/s10461-024-04471-3 (PMC11739194; doi:10.1007/s10461-024-04471-3)

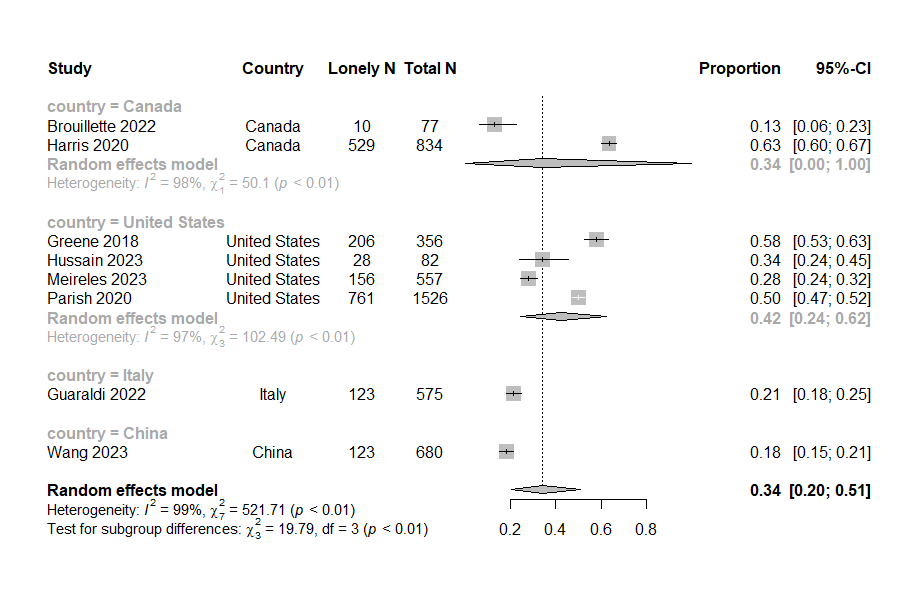

Supplement: Supplementary file 2 — Supplementary file2 (PNG 21 KB) [file 10461_2024_4471_MOESM2_ESM.png]
